# Designing the Composition of Cement-Stabilized Rammed Earth with the Association Analysis Application

**DOI:** 10.3390/ma14061390

**Published:** 2021-03-12

**Authors:** Wojciech Rogala, Hubert Anysz, Piotr Narloch

**Affiliations:** Institute of Construction Engineering, Faculty of Civil Engineering, Warsaw University of Technology, 00-637 Warsaw, Poland; w.rogala@il.pw.edu.pl

**Keywords:** cement-stabilized rammed earth, rammed earth, association analysis, market basket analysis, rule finding, rule extraction, data exploration

## Abstract

The main advantage of the structural composite material known as cement-stabilized rammed earth (CSRE) is that it can be formulated as a sustainable and cost-saving solution. The use of the aggregates collected very close to a construction site allows economizing on transportation costs. Another factor that makes sustainability higher and the costs lower is a small addition of cement to the CSRE in comparison to the regular concrete. However, the low cement content makes the compressive strength of this structural material sensitive to other factors. One of them is the composition of the aggregates. Considering the fact that they are obtained locally, without full laboratory control of their composition, achieving the required compressive strength of CSRE is a challenge. To assess the possibility of achieving a certain compressive strength of CSRE, based on its core properties, the innovative algorithm of designing CSRE is proposed. Based on 582 crash-test of CSRE samples of different composition and compaction levels, along with the use of association analysis, the spreadsheet application is created. Applying the algorithm and the spreadsheet, it is possible to design the composition of CSRE with high confidence of achieving the required compressive strength. The algorithm considers a random character of aggregates locally collected and proposes multiple possible ways of increasing the confidence. They are verified through innovatively applied association analyses in the enclosed spreadsheet.

## 1. Introduction

### 1.1. Characteristic of Cement-Stabilized Rammed Earth

One way to reduce the ecological impact and costs of construction works is to increase resource efficiency by reducing the transport of materials to the construction site [1]. This helps to ensure that construction is completed efficiently and on schedule. Maintaining a certain level of material inventory minimizes the risk of delays. Cement-stabilized rammed earth (CSRE) is a response to the search for construction material with a low construction cost and low-energy demand. The CSRE technology consists of ramming with layers a mixture of moist inorganic soil with cement addition in the formwork. The soil used to erect the walls of the building usually comes from the construction site, which allows to significantly reduce the transport of building materials and to minimize the necessary supplies.

In regions of the world where the labor cost is low and the profitability of investments is determined by the prices of materials, rammed earth seems to be the ideal construction solution. The time of erecting a wall from compacted earth using the traditional method, without mechanized construction equipment, taking into account soil preparation, transport, and erection of the wall, is from 20 to 30 h/m^3^ [2]. In the developed countries, high labor costs and the time needed to erect a building have a significant impact on the construction cost. Through mechanical mixing of the soil–cement mixture, the use of a system formwork, the use of a loader to fill the formwork with the mixture and ramming with pneumatic rammers, the efficiency at the level of 2 h/m^3^ can be achieved [2]. This is significantly less than in the case of erecting masonry structures, for which the rammed earth is an excellent alternative. Moreover, the CSRE technology enables cost-effective construction of load-bearing monolithic walls in non-urbanized areas located at a greater distance from the material producers or building depots. A significant problem of using CSRE for construction is the uncertainty of the obtained mechanical properties of the material, in particular, the compressive strength [3]. It is influenced by several properties of the soil–cement mixture, such as soil grain size [3,4,5,6,7,8,9], soil mineral composition [10,11,12], amount and type of the cement used [3,5,9,13,14], dry density [3,4,6,9] and the mixture’s moisture [3,9,15,16], curing time [17]. Moreover, the method and energy of compacting of soil layers, their height, and homogeneity also have an impact on the mechanical strength [18,19]. There are many similarities in the material design of CSRE and cement-stabilized cold re-cycling mixtures used in highway rehabilitation [20]. Both materials are sensitive to the cement content and the aggregate grain size of the mixture. The change made to these ingredients may significantly change the optimum moisture content and dry density. Likewise, the mechanical properties of both materials vary notably with the material curing time.

Soil-related properties are difficult to predict under construction conditions. In [11], it is shown that not every soil is suitable for use in the CSRE technology. Soils with highly swellable minerals should not be used [12], as their presence reduces the compressive strength and also significantly affects the durability of the material, in particular, its frost resistance and linear shrinkage. Therefore, soil with such minerals should not be used in CSRE (cement-stabilized rammed earth) technology. However, it is difficult to predict their presence without laboratory tests.

For CSRE technology to be more commonly applied, it is necessary to develop a tool that would allow engineers to predict, with a high level of confidence, the value of the compressive strength that could be obtained from the locally available soil. The authors conducted compressive strength tests on 582 CSRE samples from soils varying in their grain composition, cement content and mixture moisture. Based on the obtained results, with the use of association analysis, the algorithm is proposed to assess the confidence level of achieving the required CSRE compressive strength.

### 1.2. Standards for Rammed Earth

Currently, standards for rammed earth exist in over a dozen countries around the world. In addition, standards for this material are withdrawn rather than updated in some developed countries. For example, standards for unstabilized [21] and cement-stabilized rammed earth [22] issued in Poland in the 1960s were withdrawn some years later. According to the Polish Committee for Standardization, they could contain outdated technical data. On the other hand, in recent years, many construction standards for raw earth materials were published in many countries all over the world. It proves the growing interest in using raw earth for construction purposes [23].

The most common criteria given in the standards for the rammed earth technique are compressive strength and soil parameters, including granulation, organic substances content, soluble salts content, plasticity (Table 1). The standards provide different criteria for the durability of the material, depending on the climate conditions.

The compressive strength taken for structural calculations must be lower than characteristic compressive strength for safety reasons. On the other hand, standards suggest that the CSRE compressive strength value to be taken for calculations should be several times lower than the compressive strength obtained by specimens in laboratory tests (Table 2). 

Such difference in the results arises mainly from the very high sensitivity of compressive strength of CSRE caused by the kind of soil. As a result of such low compressive strengths for structural calculations, CSRE is rarely used in multi-story buildings. At today′s level of technology, it is possible to introduce tools that increase confidence in the mechanical strength obtained.

The aim of the article is a systematic approach to extract information from the collected large dataset comprising compositions of CSRE samples. Their compressive strengths are checked in a crush test. There are 582 samples with known aggregate composition, cement addition, water content, density and achieved the compressive strength. The association analysis seems an ideal tool to extract rules on how to compose the mixture of cement-stabilized rammed earth to achieve the required compressive strength of rammed and hardened construction material. This large database of results of analyses of CSRE samples enabled the application of a data-mining tool to design the composition of this construction material. The random character of aggregates, which are mostly collected near a construction site, causes the random character of CSRE mechanical properties. This problem can be overcome in two ways. Each batch of CSRE’s ingredients can be precisely tested in a laboratory. Then, an advanced method of designing the composition can be applied. This set of activities is not available for the builders, or it may significantly reduce the low-cost advantage of CSRE. Another possibility is to rely on the created dataset and on the results from the association analysis. To make this approach applicable, the calculator based on a spreadsheet is created and enclosed in the article. A simple sieves’ analysis made to locally dug aggregates and its results inputted to the calculator (by a builder at a construction site) is the sufficient base to design the composition of CSRE with the high confidence of achieving the required (by the builder) compressive strength of CSRE. The proposed, innovative method of CSRE designing is introduced in the article in the following sequence: the process of preparing and testing the samples is described. Then, the basis of the association analysis is introduced in Section 2. Following that, in Section 3, the calculations are made. They lead to formulating the innovative algorithm of CSRE designing with the use of the association analysis. Based on that, the calculator is created, and its functionalities are presented. How to overcome the random character of aggregates (and following that, the random character of CSRE compressive strength) with the use of the calculator is presented and discussed in Section 4. Finally, the innovative CSRE designing method with the created calculator is concluded in Section 5.

## 2. Materials and Methods

### 2.1. Database for the CSRE Samples

**Figure 1:**
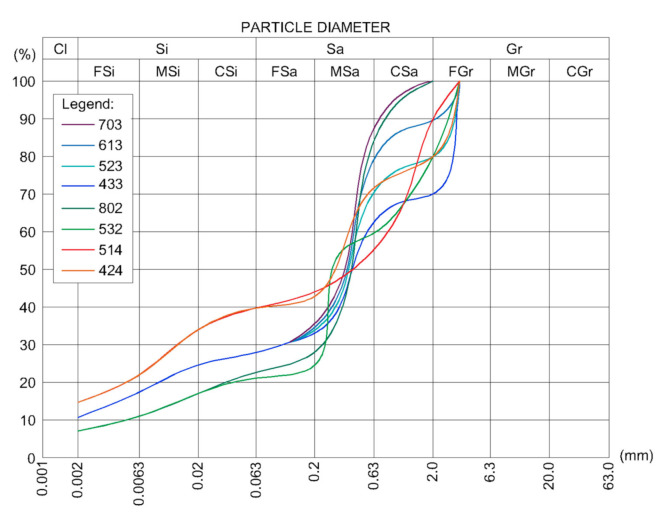
Particle size distribution of soil mixtures used for sample preparation.

Compressive strength tests were carried out on cubic samples 100 mm × 100 mm ×100 mm of soil mixtures with various amounts of cement. The particle size distribution curves of the soil mixtures used to prepare the samples are shown in Figure 1. Those mixtures were obtained by mixing silty clay with sand and gravel. Each soil mixture was named numerically to its sand: gravel: silty clay ratio by weight. For example, 10 kg of mixture 523 contained 5 kg of sand, 2 kg of gravel, and 3 kg of silty clay. For each of those mixtures, 0 to 10% by weight of Portland cement CEM I 42.5R (Odra Cement Plant, Opole, Poland) was added. Various amounts of water were added to the soil–cement mixtures, ensuring higher or equal or lower moisture content than the optimum one.

Since the mineral composition of the soil mixture, in particular, the content of clay minerals has an impact on the compressive strength of CSRE, the mineral composition of silty clay, sand and gravel was determined in a laboratory using thermogravimetric analysis. In terms of mineral composition, sand was composed of pure quartz, and gravel was 75% quartz and 25% carbonate crumbs by mass. Silty clay mineral composition is shown in Table 3 [24]. As the silty clay content in the soil mixture prior to cement addition was from 20% to 40%, the content of swelling minerals (beidellite) was from 1.8% to 3.6% of the dry weight of the mix.

**Figure 2:**
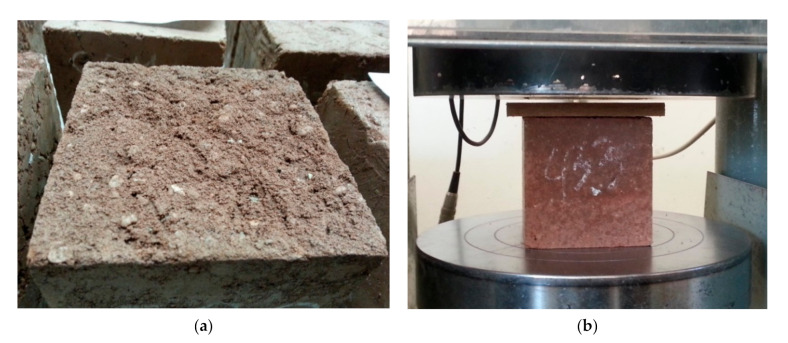
(**a**) Rough top surface of the rammed earth sample, (**b**) the sample during compressive strength test—a soft fiberboard shim was placed on the upper surface of the sample.

**Figure 3:**
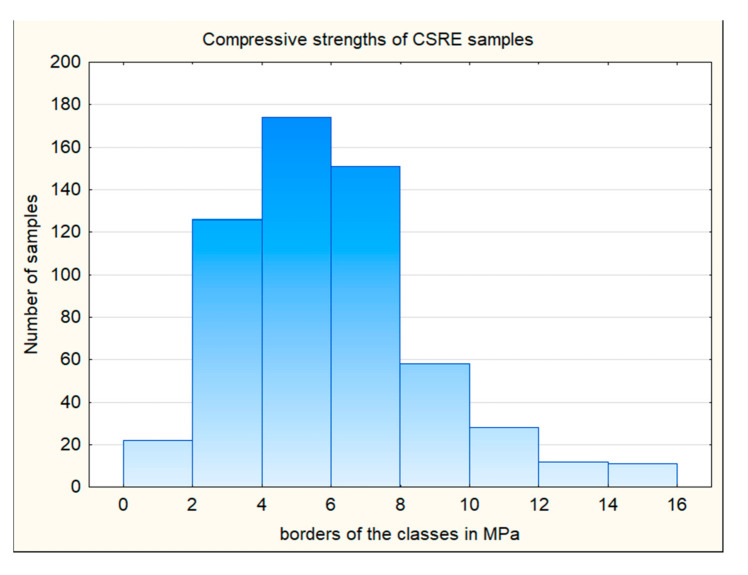
Cement-stabilized rammed earth (CSRE) compressive strength histogram

**Figure 4:**
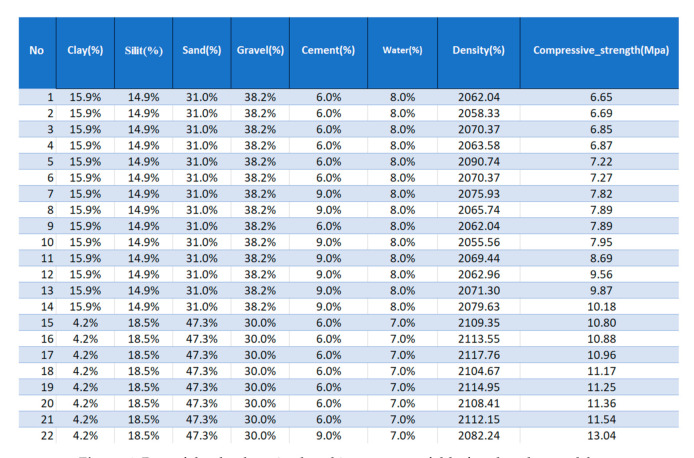
Part of the database is placed in a separate fold of enclosed spreadsheet.

The samples were formed in three layers by freely lowering the 6.5 kg rammer from a height of 30 cm to the surface of the moist soil–cement mixture. The samples to be tested were demolded after 24 h, and then they were cured for 27 days in a condition of relatively high humidity of 95% (±2%) and temperature of 20 °C (±1 °C). Since the rammed earth keeps the layered structure, the samples were loaded in the direction of ramming (Figure 2). As the surfaces of the samples were not perfectly smooth, soft fiberboard washers (Przedsiębiorstwo Handlu Drewnem i Płytami “Siekierki” s.c. Lesiewski i Synowie, Warszawa, Polska) were used. From the prepared samples, 582 compressive strength results were obtained. The results of the compressive strength are presented in the histogram (Figure 3), and the detailed results are included in Table S1. The results obtained served as a database for calculations using the association analysis application (prepared in Excel for Office 365 MSO, 2021, Microsoft Comporation, Redmond, Washington, WA, USA). The part of the database is presented in Figure 4.

### 2.2. Association Analysis

The association analysis is also called a market basket analysis [40] as it was invented to raise the sales of supermarkets. The content of the clients’ baskets was analyzed to find the rules, what goods appear in the basket jointly. Knowing that price adjustment or placing the shelves in the shop can be made, aimed at increasing the sales [41]. Let us denote *B* as a phenomenon that can be described by the states (values) of *m* parameters $\left(b_{1},b_{2},\ldots ,b_{m}\right)$and *H* as an analyzed one. The appearance of a specific *B* may trigger the appearance of *H*. Then, the rule “if *B* than *H*” can be written B→H The possibility of simultaneous appearance of *B* and *H* can be described by two ratios called confidence (*conf*) and support (*sup*) [42,43,44] (Equations (1) and (2)).

(1) $$conf\left(B\to H\right)=\frac{n\left(B\to H\right)}{n\left(B\right)}$$

(2) $$\sup\left(B\to H\right)=\frac{n\left(B\to H\right)}{N}$$

where:

n(*B→ H*)—number of cases where B and H appear simultaneously;

n(B)—number of cases of B appearance;

*N*—total number of observed cases.

The predecessor of the rule B→H , i.e., *B* is often called a body or an antecedent. The consequent *H* is often called ahead of the rule. If the confidence of the rule found is 100%, it means that every time *B* appears, *H* appears as well. However, a confidence of the rule equal to 100% can also be for the case where *B* appears only one time among *N* observations. Comparing the importance of the above-mentioned 1-case rule to the case of appearing *B* and *H* 10 times jointly, it can be said that the importance of the second rule is 10 times higher. The ratio called support helps to assess the importance of the rule (see Equations (3) and (4))

(3) $$\sup\left(B^{\left(1\right)}\to H^{\left(1\right)}\right)=\frac{1}{N}$$

(4) $$\sup\left(B^{\left(10\right)}\to H^{\left(10\right)}\right)=\frac{10}{N}$$

Nevertheless, another set of data can be created for, for which the rule *B* *→ H* has the same values of confidence and supports, but despite the same total number of observations *N*, the conclusions based on the rule will be different (see Table 4).

This is why the ration called the lift is introduced and defined as (Equations (5) and (6)):

(5) $$lift\left(B\to H\right)=\frac{conf\left(B\to H\right)}{P\left(H\right)}$$

where:

(6) $$P\left(H\right)=\frac{n\left(H\right)}{N}$$

and

*n(H)—*number of all *H* appearances in a dataset.

**Figure 5:**
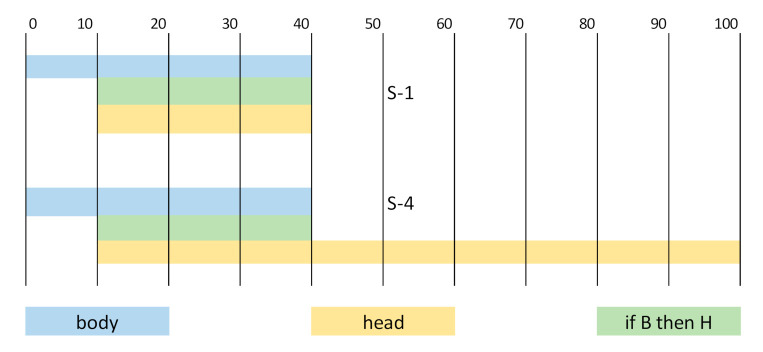
Graphical presentation of the rules with identical *sup* and *conf*, but different *lift* values.

A high probability of a head appearance *P(H)* lowers the lift. Greater than the confidence makes the lift lower than 1. A lowered lift (especially below 1) does not lower the importance of the rule *B→ H,* but it suggests that the head may depend more on other factors than the factors that were considered to describe the body. The following Figure 5 (based on [45])—where the sets of observations S-1 and S-4 are illustrated—makes the meaning of the ratios describing an association rule easier.

The described above three ratios calculated even for large datasets enable to explain the reason-effect relationship between assumed bodies and heads. Finding them contributes to predictive features of association analysis. It was utilized in multiple disciplines such as:

- Meteorology, e.g., for rainfall predictions [46];
- Biology [47,48,49];
- Medicine [50]
- Social sciences [51];
- Sales [41];
- Insurance industry [52];
- Quality management [53];
- Construction risk assessment [54];
- Bid-rigging detection [55].

This kind of analysis is especially popular in traffic safety issues. The numerous examples of market basket analysis for traffic safety assessments can be found [56,57,58,59,60]. Based on the association analysis, an algorithm for designing CSRE was invented. For easy application, a calculator based on a Microsoft Excel spreadsheet was created. The full, explored dataset is included in one of the spreadsheet folds.

## 3. Results

### 3.1. Algorithm of CSRE Designing

The core idea was to examine a locally dug-up soil, prepare its granulation analysis, and—based on the results of crushing 582 samples—assess the possibility of achieving a certain compressive strength of CSRE (of prepared with the use of the locally dug soil). The general form of the analyzed rule—based on 582 rows database—is if *B* than *H*. It is to check how likely a certain composition of CSRE supports achieving chosen compressive strength of hardened construction material. The body of the rule can be formulated as (Equation (7)):

(7) $$B=\left(b^{\left(a\right)}\cap b^{\left(c\right)}\cap b^{\left(w\right)}\cap b^{\left(d\right)}\right)$$

where:

*b^(a)^* the set of conditions concerning aggregates used for the CSRE mixture;
*b^(c )^* condition concerning the cement content in the CSRE mixture;
*b^(w)^* condition concerning the water content in the CSRE mixture;
*b^(d)^* condition concerning the achieved density of rammed CSRE mixture.

The body of the rule is equal to 1 (or its value is: *true*) where all conditions concerning the composition of CSRE (i.e., *b^(j)^ for j* = *a, c, w* or *d*) are met jointly. Otherwise, it is equal to 0 (or its value is *false*). The head of the rule (*H*) is defined as *true* (or 1) for the cases where the following inequity is met (Equation (8)):

(8) $$S_{i}\geq S_{0}$$

where:

Si >S_i_ the compressive strength if the *i-th* sample (*for i from 1 to 582*);
S0 >S_0_ the required compressive strength set by a designer.

The whole dataset is searched for the samples for which the following rule is met (Equation (9)):

(9) $$\left(B=true\right)\to \left(H=true\right)$$

The rule (9) is denoted as *B→ H.* Having the number of samples for, which the rule is met *n(B* *→ H)*, the number of samples for, which *B* is *true* denoted *n(B),* the number of samples for, which *H* is *true* denoted *n(H)*, as well as the total number of samples *N* = 582, it is possible to calculate *sup, conf*, and *lift* for the assumed mixture and the chosen compressive strength of CSRE.

#### 3.1.1. Aggregates

The majority of advantages of the application of cement-stabilized rammed as a construction material are based on the fact that the aggregates are dug close to the construction site. This implies a random character of the composition of the aggregate. Moreover, the earth material may vary in different parts of a quarry, or it can be dug in different small quarries. Also, considering the limited number of combinations of aggregates (even among 582 created and tested samples), it is assumed that each of the granulation of the examined soil will be given as a range. Minimum and maximum for clay, silt, sand, and gravel are given in Table 5.

The sum of minimum content for these 4 aggregate groups should be lower than 100%, and the sum of maximum content should be higher than 100%. If the local soil has the granulation out of the ranges presented in Table 5, the method will not produce any result. For the exemplary ranges for the local soil (clay 5 to 15%, silt 20 to 25%, sand 40 to 60%, gravel 20 to 35%), *b^(a)^* can be written as Equation (10):

(10) $$b^{\left(a\right)}=(\left(5\%\leq clay\leq 15\%\right)\cap \left(20\%\leq silt\leq 25\%\right)\cap \;\cap \left(40\%\leq sand\leq 60\%\right)\cap \left(20\leq gravel\leq 35\%\right))$$

**Figure 6:**
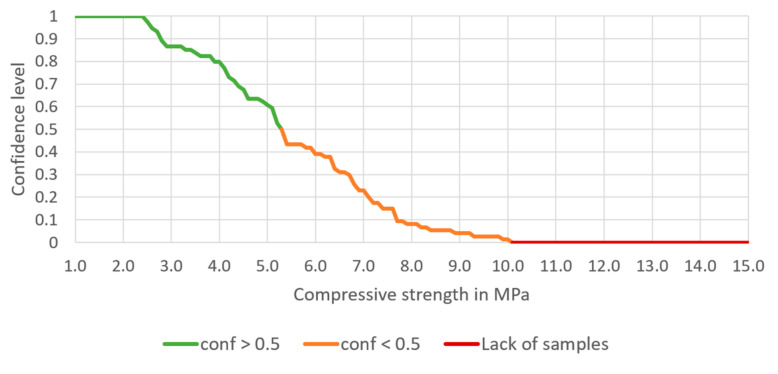
The dependence of the confidence of the rule on the chosen compressive strength, based on a selected aggregate mixture.

If the required compressive strength is set as 5 MPa, rule *b^(a)^* *→ H* can be analyzed. Based on a 582-row dataset, the confidence of this rule is 0.608, support is 0.077, and the lift is 0.964. There are 45 samples for which the conditions of the rule are met. The dependence of the confidence on the chosen compressive strength is presented in Figure 6.

It can be noted that there is 100% confidence of achieving 2.4 MPa of the compressive strength when this type of aggregates mixture is used, and there is no sample when the compressive strength above 10 MPa is achieved (for this aggregate mixture).

#### 3.1.2. Cement

For all 582 samples only 4 level of cement are used, i.e., 0%, 3%, 6%, 9%, and 10%. The designer is allowed to use only those values. For exemplary 6% addition of cement, it can be denoted that Equation (11):

(11) $$b^{\left(c\right)}=6\%$$

For all prepared samples, only one type of cement, CEM I 42.5R, is used. Then the rule based on aggregates given in Section 3.1.1 and 6% cement addition, i.e., considering Equations (10) and (11), can be formulated as

(12) $$\left(b^{\left(a\right)}\cap b^{\left(c\right)}\right)\to H\left(\min5MPa\right)$$

**Figure 7:**
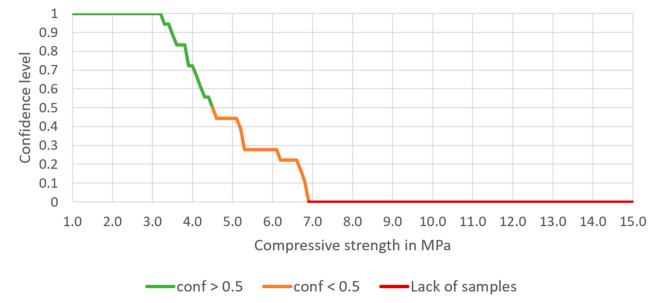
The dependence of the confidence of the rule on the chosen compressive strength, based on selected aggregate mixture and 6% cement content.

The confidence of the rule (Equation (12)) is 0.444, the support is 0.013, and the lift is 32.333. There are 8 samples meeting this rule. For the rule (Equation (12)), the dependence of the confidence on the chosen compressive strength can be drawn (see Figure 7).

The addition of 6% cement extended the range of the compressive strength achieved with 100% confidence up to 3.2 MPa. However, there is no sample prepared with the assumed mixture and 6% cement addition for which the compressive strength is higher than 6.9 MPa.

#### 3.1.3. Water Content

The next step of the designing procedure is to select the water content in the CSRE mixture. Similar to cement content, the water content during the preparation of the samples is applied in a step manner. Only 9 levels of water content can be found from 6 to 14% (with the step 1%). Assuming—for example—the water content of 11%, it can be formed (Equation (13)):

(13) $$b^{\left(w\right)}=11\%$$

For the rule (Equation (14)):

(14) $$\left(b^{\left(a\right)}\cap b^{\left(c\right)}\cap b^{\left(w\right)}\right)\to H\left(\min5MPa\right)$$

**Figure 8:**
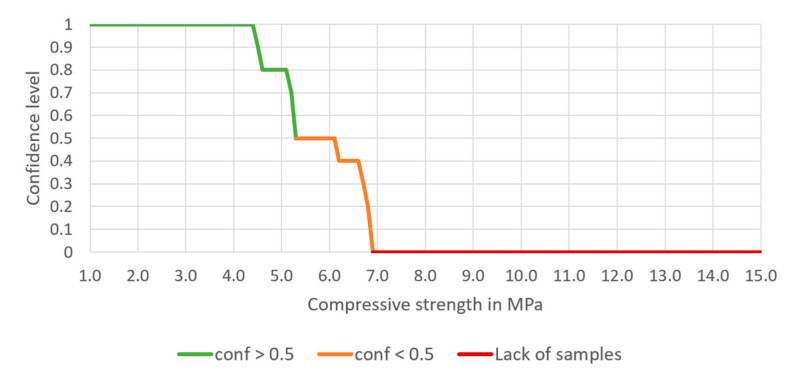
The dependence of the confidence of the rule on the chosen compressive strength, based on selected aggregate mixture and 6% cement content, and 11% water content.

The confidence is now 0.800, the support equals 0.014, and the lift is 58.2. The confidence for different compressive strengths set as a minimum for the rule (Equation (14)) is presented in Figure 8.

This time, making the set of conditions of the predecessor even more specific increases the confidence of achieving 5 MPa significantly, to 80%. Furthermore, the lift increases to 58.2, suggesting that 5 MPa and higher can be explained by the present conditions of the predecessor. Moreover, for this set of conditions, the compressive strength up to 4.3 MPa is achievable with 100% confidence.

#### 3.1.4. Density

This parameter of the CSRE mixture represents the energy used for ramming. The density depends on the aggregates (their granulate composition, the volume weight of the material they are built of), and it also depends on the water content in a mixture (and cement content too). However, for a certain mixture, its density depends mainly on the energy extent used for the compaction process—ramming the mixture. The more energy spent on ramming, the higher the density is (up to the given maximum). As the prepared 582 samples of CSRE had different densities, the density is set as the next condition of the predecessor of the rule. The exact value in kg/m^3^ with the range ±50 kg/m^3^ should be given as an input. The range is a compromise between the full range of densities (for all 582 samples), i.e., from 2054 to 2406 kg/m^3^ and the number of samples being covered, while a specific density is chosen. If, for example, 2100 kg/m^3^ is chosen, then it can be written Equation (15):

(15) bd=2050kgm3≤density≤2150kgm3

Then the examined rule has the from Equation (16):

(16) $$\left(b^{\left(a\right)}\cap b^{\left(c\right)}\cap b^{\left(w\right)}\cap b^{\left(d\right)}\right)\to H\left(\min5MPa\right)$$

Its full form (considering equations (10), (11), (13), and (15)) can be denoted as Equation (17):

(17) [5%≤clay≤15%∩20%≤silt≤25%∩40%≤sand≤60%∩∩20≤gravel≤35%∩6% of cement ∩11% of water content ∩∩2050kgm3≤density≤2150kgm3]→Hmin5 MPa

**Figure 9:**
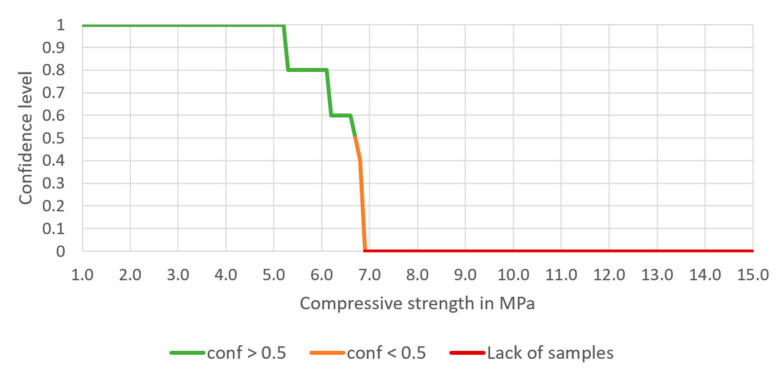
The dependence of the confidence of the rule on the chosen compressive strength, based on selected aggregate mixture and 6% cement content, and 11% water content, and 2100 ± 50 kg/m^3^ density of CSRE.

For the rule (stated as Equations (16) and (17) comprising all the ingredients for the CSRE and its density, the confidence is 1.000, the support is 0.009, and the lift is 116.400. The rule is met for 5 samples. If all conditions comprised by the predecessor are met (it happened 5 times among 582 created samples), then every time, the achieved compressive strength of the sample is 5 MPa or higher. It can be observed in Figure 9.

#### 3.1.5. The Flowchart of the Designing Process

**Figure 10:**
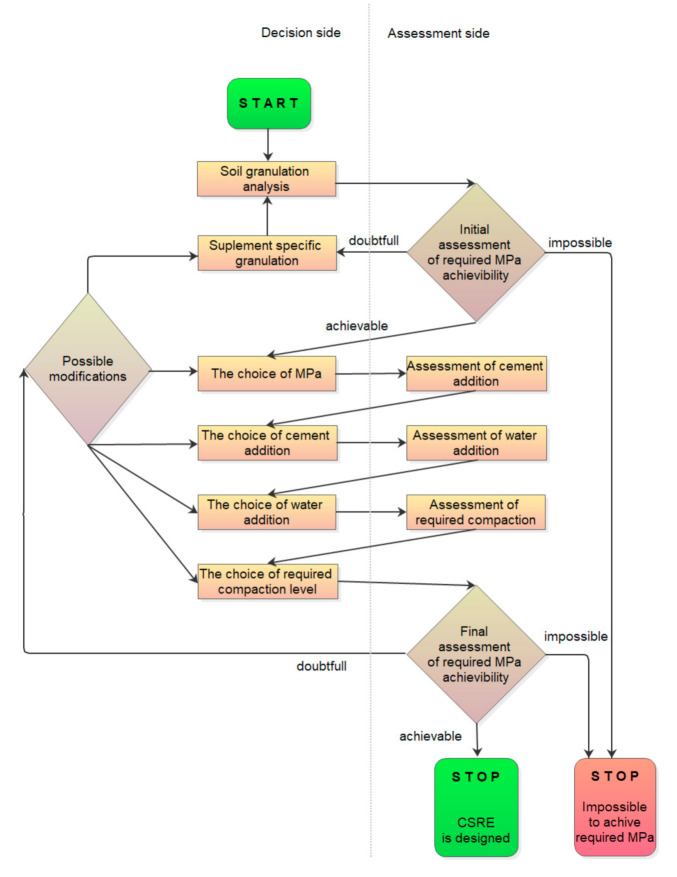
Proposed algorithm for designing CSRE.

As the algorithm (Figure 10) is created for site application of CSRE structures, the natural start concerns the check of soil granulation. Economizing by the use of cement-stabilized rammed earth requires utilization of the soil locally dug for construction purposes. Together with the sieves analysis, the natural water content should be checked. The water content in the presented algorithm comprises the sum of water (existing naturally in the soil together with added to a mixture amount of water).

### 3.2. Calculator for Designing CSRE Based on Association Analysis

Three tools are analyzed for the implementation of the algorithm— Structured Query Language (SQL) database with website interface, the Visual Basic for Applications (VBA) programming interface in Microsoft Excel and a standard Microsoft Excel spreadsheet.

SQL database seems to be the best tool as a final solution. The online database is more scalable—each database extension is seen for all users without the need for file updates. What is more, each SQL inquiry can be registered, and this could help to determine the most popular mineralogical compositions and to detail the database to the most reasonable extent. Nevertheless, it is the most expensive solution, which requires external IT services supplier.

The VBA application programming interface in Microsoft Excel was developed to extend the standard functionality of a workbook. It was used by authors, e.g., for simulation purposes in the research [61,62]. The use of VBA for the CSRE designing allows not only for searching for samples meeting the rules but also for listing them. The sample code used for finding the samples is presented below.

(18) $$Range\left(“\$A\$1:\$X\$583”\right).AutoFilter\;Field:=2,\;Criteria1:=“>=“\;\&\;Arkusz2.Cells\left(6,\;4\right)\;*\;100\;\&\;“\%”,\;\_Operator=xlAnd,\;Criteria2=”\leq ”\;\&\;Arkusz2.Cells\left(6,\;5\right)\;*\;100\;\;\&\;“\%”$$

where:

*(“$A$1:$X$583”)*—is the range of database;

*Field:= 2*—number of columns, where the values are filtered (clay in this case);

*Criteria1:=”>=” Arkusz2.cells(6,4)*—only the records higher or equal to the value specified in particular cells will be shown;

Criteria2:= analogy to criteria1;

Operator:=xlAnd—all criteria listed in the code must be filled.

The disadvantage of the *.xlsm files (files using VBA code) is that the file can be opened only by the desktop version of Microsoft Excel. It can be open neither via browser version nor via free of charge *.xlsx files editors. Due to that, this solution was not further developed.

MS Excel is one of the most popular applications. It can be assumed that the *.xlsx editor is available on almost every personal computer (delivered by Microsoft or another supplier). Therefore, the authors decided to use this tool for CRSE designing. The only disadvantage of such a solution is having shared the file, the control of the file is lost. Using tools available in Office 365, like OneDrive, partially eliminate this disadvantage.

The first step of designing a tool for handling the database queries is to design the database in optimal form. During the backend designing, the good practice is to place the database in a separate fold and to name the headings.

The database queries for market basket analysis often consists of dozens of conditions. Standard *.xlsx spreadsheet does not allow to place the comments for parts in formulas, as well as to break the lines. Without naming the ranges, it is nearly impossible to locate the mistake or modify the formulas. This problem can be solved by defining the database as a table (insert tab-> table). Due to this operation, each column is named, which makes the formulas understandable.

**Figure 11:**
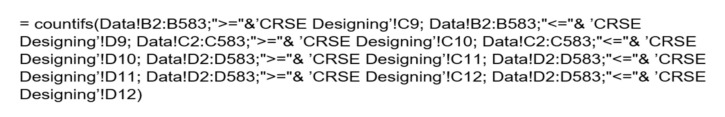
Example of Excel formula used before naming the ranges and using tables.

**Figure 12:**
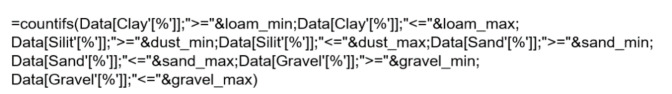
Example of Excel formula used after naming the ranges and using tables.

Another step that facilitates the navigation through the table is naming the cells that are frequently used. The difference between standard database and database where MS tables and naming the ranges were used is shown in Figure 11 and Figure 12.

A standard Microsoft Excel spreadsheet allows designing a user-friendly interface. By a few simple measures, the visual interface of the tool based on an MS Excel sheet can completely differ from the standard appearance of an application.

Having designed the interface of an application, all sheets, which are not edited by the user, must be hidden. The visual appearance is much improved after hiding rows and columns, which are not used (below and the right from the user interface). Another measure is to hide the headings in a fold (option available in view tab). All cells except the ones that are to be edited by the user should be set as hidden and protected. The effect of this operation is seen after protecting the sheet (available in the review tab). Protection of protected the folds and the workbook secures their content from unintentional interference in formulas. It must be underlined that this option is not sufficient in the case of confidential and secret data.

Many input data must be limited to a certain range possible, e.g., by using drop-down lists.

**Figure 13:**
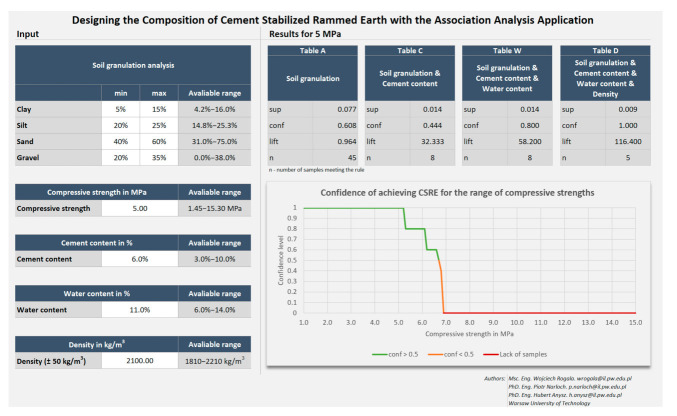
The appearance of the application.

Having taken all those measures, the visual interface looks like rather an application than a standard spreadsheet (Figure 13). Such an interface is also resistant to user modifications and random mistakes.

The market basket analysis bases on counting the records, which meet the rules by using the countifs formula, which structure is:

(19) $$=countifs\left(range_{1};criteria_{1};\ldots ;range\_n;criteria\_n\right)$$

To start working with the calculator, the composition of the aggregate of the soil must be entered. In addition to each aggregate type (clay, silt, sand, gravel), the range is covered by existing test results is presented. It is impossible to enter the data out of this range.

Having entered the minimum and maximum contents of each aggregate, the expected compressive strength should also be entered. Having finished this step, confidence, lift and number of samples meeting the rules appear in Table A. The chart presented below Table A shows the potency of the cement-stabilized rammed earth, based on the composition of the aggregate. Thus, a confidence level can be read for each compressive strength (1.0 to 15.0 MPa). There are three series on the chart—the green one presents the compressive strength with a confidence level higher or equal to 50%, the orange one—the compressive strength with a confidence level lower than 50%. The rest values are marked in red, which means that the database does not include samples with such compressive strength.

In the next step, the cement content is to be designed. Having chosen the cement content between 0% and 9%, the association analysis ratios appear in Table B. The two last steps concern is designing the water content and the density, as per the previous step.

The Excel graphical user interface is considered the best tool. It is commonly available and could be adapted without external suppliers. It allows to design walls made of cement-stabilized rammed earth without detailed knowledge of the market basket analysis. The description of the formulas applied allows using the market basket analysis also for other research.

## 4. Discussion

### 4.1. General Findings

The technology of preparing cement-stabilized rammed earth differs greatly from the technology of widely applied concrete. The main differences are presented in Table 6.

**Figure 14:**
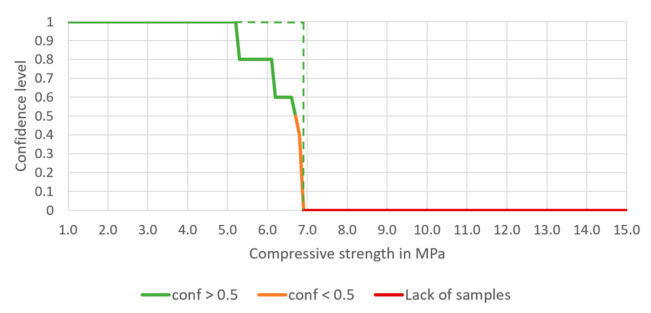
Ideal dependence of confidence of achieving certain compressive strength of CSRE (green dotted line) based on a certain prescription.

Having compared these two construction materials, it is clear that in the case of concrete, if a certain mixture composition is used in the production process (and the technological regime is respected while placing a concrete mixture), there is practically no doubt that the designed compressive strength is achieved. However, even then, for load-bearing structures, several samples are prepared on-site to verify the compressive strength in a laboratory. The confidence of achieving the designed compressive strength—for such a well-known construction material as concrete—would look like the green dotted line in Figure 14 if a recipe for achieving 6.9 MPa is used.

**Figure 15:**
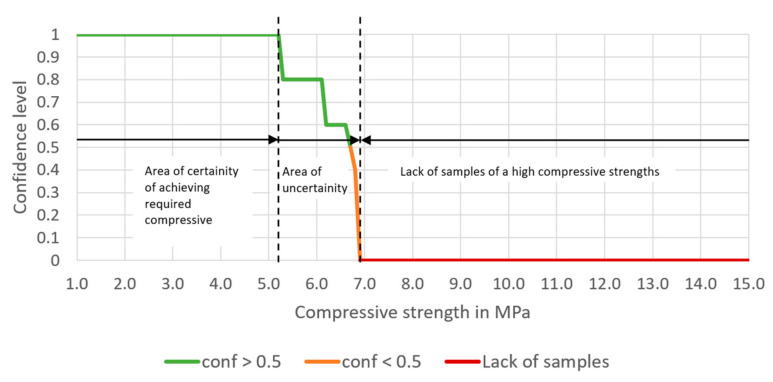
Areas of different certainty of achieving certain compressive strength.

The processes of preparation and building-in the CSRE have much more random character. The proposed design of the cement-stabilized rammed earth with the use of association analysis is based on structural analysis of 582 prepared samples of different sets of ingredients, i.e., it is also an experience-based process. Having the conditions added to the body of the rule, it can be observed in Figure 6, Figure 7, Figure 8 and Figure 9 that:

- The range of the compressive strengths achievable with 100% confidence increases (to the higher values; the horizontal part of the green line at conf = 1);
- The range of not achievable compressive strengths is extended to the lower values in the step where the condition the content of cement is set (the red line);
- These (mentioned above) made the “Area of uncertainty”—presented in Figure 15—narrower;

- The area where the confidence of achieving a given compressive strength is higher than 0 and lower than 0.5 becomes very narrow (the orange line));
- Except for the step where the cement content is set, the confidence of achieving 5 MPa rises from 60.8% to 100%;
- The number of samples meeting the rule lowers with added conditions to the predecessor (body of the rule);
- The lift of the rule rises with every condition added to the body (starting from 0.964 and ending at 116.400);
- The lowering number of samples meeting the rule (while the conditions are being added) results in lowering support of the rule;
- There are 5 samples meeting the full (with all conditions added to the predecessor) rule, i.e., the compressive strength for them is 5 MPa or higher. In other words, for the whole dataset, if the conditions of the predecessor are met, every time the compressive strength of the sample is 5.0 MPa or higher, as the conf = 100%.

**Figure 16:**
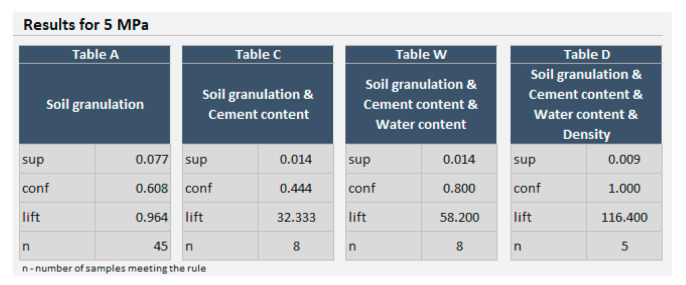
The screenshot of the calculator. Parameters of the rules for achieving 5 MPa presented for different predecessors (bodies).

These effects can also be observed on the right part of the calculator, as presented in Figure 16.

It can be observed how the certainty of achieving the required compressive strength rises with every step, i.e., with every condition added to the original predecessor (the set of soil granulation). The exception is the condition for the cement content, but even then—despite the confidence lowers to 0.444—the lift of the rule increases high above 1.0 (reaching 32.333). This ensures the user of the calculator that the confidence of the rule concerns the majority of the samples from the dataset with the compressive strength equal to or higher than 5 MPa. Referring to the algorithm of designing (presented in Figure 10), it can be stated that the intermediate assessments are helpful for the designer of the CSRE mixture, but the final assessment is the most informative.

### 4.2. The Procedure of Designing CSRE

The procedure starts with examining the soil dug close to the construction site and inputting its granulation to the calculator. The initial assessment—which is based only on soil granulation—is the first critical point. The path marked as “impossible” should be chosen only if there is a lack of samples for the chosen compressive strength. Observing Figure 6, it can be found that, for the exemplary granulation (described in Section 3.1.1), achieving more than 10.0 MPa is impossible for this soil. The designing process should be stopped then, or the required compressive strength should be lowered. It can be read in this figure that for 50% of samples made of this soil (i.e., the soil with similar granulation), the achieved compressive strength is approx. 5.2 MPa (if entered into the calculator, the exact value is 5.21 MPa). It is to emphasize that this is independent of cement and water content and independently from the density achieved. Hence, even if confidence is 50% (when only granulation is used as a predecessor), achieving 5 MPa is not doubtful. The path “doubtful” during the initial assessment (in the algorithm) should be chosen only if the confidence of achieving the required compressive strength is really low (below approx. 10–15%. If it is chosen, then enriching the soil with a specific size of granulation should be considered. The size can be found with the use of the calculator by modifying the ranges of soil. If the confidence for the original soil is over 10–15%, the path “achievable” should be chosen, and dealing with cement and water content, the maximum of confidence should be searched (in table W of the calculator; table C there serves as an auxiliary at this stage of designing). Having the conditions (cement and water content) providing the maximum confidence found, it must be checked (with the use of a calculator) what range of densities make the confidence—in table D of the calculator—higher than in table H there. If it is not possible, the confidence in tables H and D should be at least equal. If the confidence in table H is 100%, it means that for all samples meeting the conditions of the predecessor, the achieved compressive strength is equal to or higher than required. The number of samples meeting the rule can be read in the calculator. Then, the real test must be made to check if it is possible to achieve the required density of the CSRE mixture in the process of ramming the mixture. If yes, CSRE is designed.

**Figure 17:**
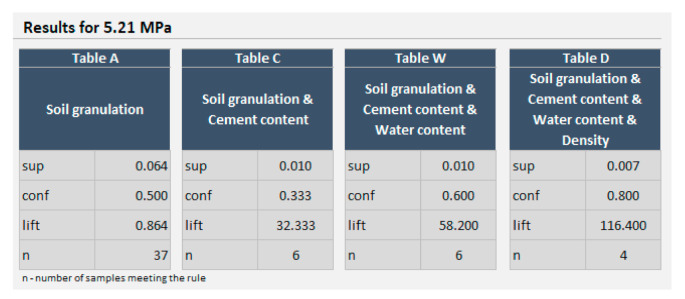
The result from the calculator for 5.21 MPa.

It may happen that 100% confidence in table D (in the calculator) is not reached. Let us discuss it for the 5.21 MPa set as a required one (the other predecessor conditions are as before). The result from the calculator is presented in Figure 17.

The final confidence (i.e., in Table D) is 80%. It can be shifted to 100% by increasing the density to 2140 kg/m^3^. Nevertheless, there is only one sample meeting the rule. The confidence is 100%, so there is also one sample (the same one) meeting the criteria of the predecessor. For a single sample, the compressive strength could be reached by chance. Another way of reaching 100% confidence in Table D is to change the cement content and adjust the water content then. For 9% of cement, 10% of water content, 2100 kg/m^3^ density (aggregates unchanged), the confidence is 100% again, with two samples meeting the rule. The figure is drawn in the calculator then provides the user with the information that 100% is even for 5.9 MPa. The same can be read in Table D when 5.9 is entered as input. If the user does not like giving up originally assumed 5.0 MPa, and the aforementioned changes do not bring the increase in the final confidence, the changes in granulation can be considered. The calculator allows for experiments with soil granulation, but in fact, it is not recommended. Delivering a specific fraction of aggregates to a construction site can cancel economizing by the use of CSRE (also, the need to mix the delivered fraction of aggregates with original soil increases the labor intensity and cost). Second, narrowing the ranges of the content of a specific fraction may lower the support of the rule or even do not give results from the calculator.

Considering all the above, the outlets from the final assessment node (see Figure 10), i.e., “achievable”, “doubtful”, “impossible,” should be chosen for parameters of different levels than for the initial assessment node. Here, it is strongly recommended to accept 100% confidence to state that the required compressive strength can be achieved. The authors’ use of the calculator proves that it is difficult to achieve the highest confidence (in Table D) by modifying other parameters if the originally calculated confidence is below 80%. As there are many possible combinations, they are not checked. Hence, the precise lower limit of confidence for the path “doubtful” in the final assessment node cannot be given. Certainly, the confidence of reaching the required compressive strength below 60% in this node cannot be accepted. If, for example, conf = 60% and n = 12 (presented in the calculator), it means that there are 20 samples (12/0.6) meeting the conditions of the predecessor, but only 12 of them have the required compressive strength or higher. Hence, it is impossible to achieve the assumed compressive strength with high confidence, and just this “impossible” path should be chosen (which leads from the final assessment node to the stop of designing procedure with no mixture designed). In this case, it can be proposed to redesign the structure, which can make the required compressive strength lower, possible to achieve with the highest confidence, and based on locally dug soil.

### 4.3. The Assessment of the Proposed Method

The proposed method based on results of 582 tests, enhanced by association analysis, allows to verify if the kind of inorganic soil found close to a construction site can serve as an ingredient of CSRE for a structure, where a certain compressive strength is required. The only physical tests that must be done are checking the soil granulation, initial soil humidity and the density achieved for the designed CSRE mixture. Based on them, with the use of the proposed calculator, many variants of CSRE components can be checked to find the best composition providing 100% confidence in achieving the desired compressive strength. The advantages of the proposed method can be listed as:

- The ease of use (only the aforementioned simple physical tests and spreadsheet with the calculator are needed);
- The quick result;
- The objectivity of the result (as it is the experience-based method);
- The clarity of the result (if 100% confidence is achieved, it is known that there is a specific number of similar samples in the database, and for all of them, the compressive strength is not lower than assumed in the designing process);
- The confidence for the whole ranges of the compressive strength can be observed in the figure built in the calculator (it allows for instant assessment of what level of the compressive strength is achievable with 100% confidence)
- The time (and cost) saving (the use of the calculator allows experimenting with proportions of ingredients and instant control of CSRE parameters);
- The extendibility (more laboratory test results can be added to the database for more precise and more certain results).
- Nevertheless, the method has some limitations as:
- The lack of possibility of making the ranges of specific aggregates very narrow (it may produce a lack of samples in the database and then no result);
- It is possible (in the calculator) to apply only specific values of cement content and only specific values of water content (only these values are used to prepare samples in the database). It is not the limitation of the method generally, but the limitation of the database created;
- The calculator will not produce any result if any of the ingredients content is out of the presented range (these ranges arise from the composition of the samples in the database);
- The present database cannot be extended horizontally, i.e., by adding other CSRE mixture parameters as only presented features are checked for all 582 samples. However, for the new database and the method itself, there is no limit for the number of features;
- The conditions for the predecessor should be entered with the presented (in the calculator and in the article) top-down sequence, e.g., there will be no result produced if the cement content is entered without entering the contents of the aggregate. It is the limitation of the calculator, not of the method.
- Not all features influencing the compressive strength are considered in the database as, e.g., mineral composition. The first step before choosing the CSRE technique for construction should be to determine the soil mineral composition and content of organic substances. If, as a result of these tests, it turns out that the soil contains organic matter or more swelling minerals than the soil used in the research, the authors suggest the use of a construction technology other than the CSRE.

Weighing the pros and cons for the use of association analysis for designing the composition of cement-stabilized rammed earth, it seems that it is the right way of assessing the certainty of repeatable results of the compressive strength if calculations are based on tests of hundreds of samples (of different composition). The created database is large enough to apply machine learning tools. Some of them have already been applied by the authors in [3,6,63]. The compressive strength is predicted, and an impact of CSRE ingredients on the compressive strength is searched there. The proposed algorithm is designed for the case where the ranges of the aggregate fractions serve as an input (not their exact values). The applied before machine learning tools are designed for the exact values at the input. Entering several combinations of aggregate compositions (based on ranges applied in association analysis) would produce several compressive strength predictions. The reasoning based on them about the compressive strength possible to achieve could be done, but the prediction errors—the measure of uncertainty—should be considered every time. The proposed approach, if 100% confidence is achieved, filters the database and presents the number of samples, which exactly meet the criteria (set at the input) expressed with values typical for the association analysis. The uncertainty of the invented method still exists. It is uncertain how much the exact aggregate distributions of the reference samples from the database fit the exact aggregate distribution of the soil collected at a construction site. The method does not assume this kind of verification, as it was invented (based on the scientific tools) for practical application at a construction site. However, it can be stated that the narrower the ranges inputted are, the lower uncertainty of the differences in aggregate distribution is, but it lowers the number of samples fitting the input criteria. It is to emphasize that, having considered the imperfections of the process of creating CSRE at a construction site, the information gained from the proposed method about the number of similar samples, for which the compressive strength achieved is every time no lower than a certain value, is a sufficient base for conclusions concerning the compressive strength of CSRE possible to achieve at a certain construction site.

## 5. Conclusions

The database of 582 tested samples of the cement-stabilized rammed earth—created by the authors—can serve as a reference for builders willing to apply CSRE as a construction material. The association analysis applied to the database—thanks to its simple, easily understandable ratios—allows assessing of construction abilities of soil dug at a given construction site. The random character of the dug-at-site soil made the standards for designing CSRE very conservative. Nevertheless, as presented in the article, it is possible (based on 582 rows database) to find a composition of CSRE, which provides high confidence of reaching the designed compressive strength. It must be noticed that even the 100% confidence is, based on aggregates granulations given by ranges, as well as on density given by the range (not the precise value). Hence, it may happen that the achieved compressive strength is lower than the designed one with 100% confidence. It is expected that the level of a safety coefficient (lowering the design compressive strength—the result of tests of CSRE) should be much higher than those in New Zealand and German standards. Its value for the proposed method of CSRE designing is the area of interest and will be researched by the authors. This novel approach to calculate the composition of CSRE through a data exploration—the association analysis—allows minimizing the random character of ingredients and the final features of this construction material. The algorithm is based on a simple test (that can be made on-site) and built in the Excel file. All the above make the method easy to utilize by the builders. The results of the proposed method are clearly presented in the calculator and can be understood by technical staff working on a construction site.

The calculator (Supplementary Materials Table S1) is based on the association analysis and proposed algorithm of CSRE designing. Currently, the database comprises 582 test samples of CSRE. The researchers interested in expanding this database by sharing their results (and adding them to the database) are highly welcome. Moreover, even the present form of the Excel calculator based on the innovative algorithm can be helpful for builders and researchers.

The authors realize that other mixture properties and material preparation aspects, not included in this calculator, also can have an impact on the compressive strength of rammed earth. Therefore, in the next steps, the authors foresee expanding the calculator with features that affect the compressive strength, such as the mineral composition of the soil, energy and method of mixture ramming, time and conditions of material curing. It is also planned to expand the calculator with compressive strength results carried out on mixtures of soil with more ecological binders. Moreover, knowing that the compressive strength is not the only important feature of the construction material, in further research, the expansion of the calculator with other mechanical parameters is considered.

## Figures and Tables

**Table 1:** Characteristics of rammed earth included in the standards [23,24].

Document	Ref.	Country	Properties of the Soil Mixture				Properties of the Material				
							Mechanical Strength		Durability		
			Granulation	Organic Substances	Soluble Salts	Plasticity	Compressive Strength	Tensile Strength	Linear Shrinkage	Frost Resistance	Resistance to Water
CSIRO Bulletin 5 (1995)	[25]	Australia		X			X				X
EBAA (2004)	[26]			X	X		X				
HB 195-2002	[27]			X			X		X		
Lehmbau Regeln (2009)	[28]	Germany		X	X		X		X		
IS: 2110 (1998)	[29]	India	X		X	X	X				
IS: 13827 (1998)	[30]		X			X					
PCH-2-87 (1988)	[31]	Kyrgyzstan	X		X	X	X				
NZS 4297 (1998) NZS 4298 (1998) NZS 4299 (1998)	[32] [33] [34]	New Zealand			X		X	X	X		X
14.7.4 NMAC (2006)	[35]	USA	X	X	X		X				
ASTM D 560 (1996)	[36]									X	
ASTM D559 (2003)	[37]										X
SAZS 724 (2001)	[38]	Zimbabwe	X	X	X		X				
BN-62/6738-01 BN-62/6738-02	[22] [21]	Poland	X				X		X		X
MOPT Tapial (1992)	[39]	Spain	X	X			X				

**Table 2:** Design compressive and characteristic compressive strength of rammed earth.

Country	Standard ref.	Design Compressive Strength (MPa)	Characteristic Compressive Strength (MPa)
Germany	[28]	0.3–0.5	2.0–4.0
New Zealand	[33]	0.5	The compressive strength of all tested specimens must be higher than 1.3

**Table 3:** Mineral composition of silty clay used (%).

Clay Minerals			Siderite	Quartz
Beidellite	Kaolinite	Illite
8.9	8.6	26.2	6.0	50.3

**Table 4:** The exemplary data sets with different lifts.

Set of Observations	*N*	*n(B)*	*n(H)*	*n(B* *→H)*	*sup*	*conf*	*lift*
S-1	100	40	30	30	0.30	0.75	2.50
S-2	100	40	60	30	0.30	0.75	1.25
S-2	100	40	80	30	0.30	0.75	0.94
S-4	100	40	90	30	0.30	0.75	0.83

**Table 5:** The limits of the ranges of content for each size of aggregates.

Aggregate size	Min Content	Max Content
Clay	4.2%	16.0%
Silt	14.8%	25.3%
Sand	31.0%	75.0%
Gravel	0.0%	38.0%

**Table 6:** Main differences between CSRE and concrete.

Features	CSRE	Concrete
Place of a mixture preparation	Most often on the construction site	Most often in the production plant
Set of aggregates	Dug close to the construction site	Selected fractions delivered to the production plant
Mixture composition	Adjusted to the aggregates dug and to the application requirements	Adjusted to the concrete application requirements
Ingredients batch control	On-site, by experienced builders	Quality control procedures, precise measuring instruments
Mixture consistency under construction	Loose, moist mixture	Liquid or semi-liquid mixture
Popularity of material standards	Standards in over a dozen countries around the world	Standards in most developed countries of the world

## Data Availability

The full dataset is available in the Excel file–the created calculator–and it is published as Supplementary Materials along with the article.

## Supplementary Materials

The following are available online at https://www.mdpi.com/1996-1944/14/6/1390/s1, Table S1: Calculator for the CSRE designing based on association analysis and database of CSRE samples.
